# A snapshot of the prevalence of dihydropteroate synthase-431V mutation and other sulfadoxine-pyrimethamine resistance markers in *Plasmodium falciparum* isolates in Nigeria

**DOI:** 10.1186/s12936-023-04487-5

**Published:** 2023-03-01

**Authors:** Adebanjo J. Adegbola, Omotade A. Ijarotimi, Akaninyene E. Ubom, Bukola A. Adesoji, Olajide E. Babalola, Emma F. Hocke, Helle Hansson, Andria Mousa, Oluseye O. Bolaji, Michael Alifrangis, Cally Roper

**Affiliations:** 1 Department of Pharmaceutical Chemistry, Faculty of Pharmacy, Obafemi Awolowo University, Ile Ife, Nigeria; 2 Department of Obstetrics, Gynaecology and Perinatology, Faculty of Clinical Sciences, College of Health Sciences, Obafemi Awolowo University, Ile-Ife, Nigeria; 3 Department of Obstetrics, Gynaecology and Perinatology, Obafemi Awolowo University Teaching Hospitals Complex, Ile-Ife, Nigeria; 4 Department of Nursing Services, Obafemi Awolowo University Teaching Hospitals Complex, Ile-Ife, Nigeria; 5 State Specialist Hospital, Asubiaro Osogbo, Nigeria; 6 Department of Immunology and Microbiology, Centre for Medical Parasitology, University of Copenhagen, Copenhagen, Denmark; 7 Department of Infectious Diseases, Copenhagen University Hospital (Rigshospitalet), Copenhagen, Denmark; 8 Department of Infection Biology, Faculty of Infectious and Tropical Diseases, London School of Hygiene and Tropical Medicine, London, UK

**Keywords:** SP resistance, Molecular marker, Dihydropteroate, Dihydrofolate, Malaria, Pregnancy

## Abstract

**Background:**

Malaria is a major public health issue with substantial risks among vulnerable populations. Currently, the World Health Organization (WHO) recommends SP-IPTp in the second and third trimesters. However, the efficacy of SP-IPTp is threatened by the emergence of sulfadoxine-pyrimethamine resistant malaria parasites due to single nucleotide polymorphisms in the *Plasmodium falciparum* dihydrofolate reductase and dihydropteroate synthetase genes. This study aimed to assess the current prevalence of *Pfdhfr/Pfdhps* mutations in *P. falciparum* isolates collected from individuals residing in Ile-Ife, Nigeria, and also present maps of the prevalence of *Pfdhps* 431V and 581G within Nigeria and surrounding countries.

**Methods:**

Between October 2020 and April 2021, samples were collected as dried blood spots among 188 participants who showed malaria positivity with a histidine-rich-protein-based rapid diagnostic test (RDT). Nested PCR assays were used to confirm falciparum in the samples with RDT positivity, and to amplify fragments of the *Pfdhfr/Pfdhps* genes followed by targeted amplicon sequencing. Published data since 2007 on the prevalence of the *Pfdhps* genotypes in Nigeria and the neighbouring countries were used to produce maps to show the distribution of the mutant genotypes.

**Results:**

Only 74 and 61 samples were successfully amplified for the *Pfdhfr* and *Pfdhps* genes, respectively. At codons resulting in N51I, C59R, and S108N, *Pfdhfr* carried mutant alleles of 97.3% (72/74), 97.3% (72/74) and 98.6% (73/74), respectively. The *Pfdhps* gene carried mutations at codons resulting in amino acid changes at 431–436-437–540-581–613; I431V [45.9%, (28/61)], A581G [31.1% (19/61)] and A613S [49.2% (30/61)]. Constructed haplotypes were mainly the triple *Pfdhfr* mutant 51I-59R-108N (95.9%), and the most common haplotypes observed for the *Pfdhps* gene were the IS**G**KAA (32.8%), IS**G**K**GS** (8.2%), **V**A**G**KAA (14.8%), **V**A**G**KA**S** (9.8%) and **V**A**G**K**GS** (14.8%). In the context of the previously published data, a high prevalence of 431V/581G mutations was found in the study population. It seems quite evident that the *Pfdhps* 431V, 581G and 613S often co-occur as *Pfdhps*-**V**A**G**K**GS** haplotype.

**Conclusion:**

This study showed that the prevalence of **V**A**G**K**GS** haplotype seems to be increasing in prevalence. If this is similar in effect to the emergence of 581G in East Africa, the efficacy of SP-IPTp in the presence of these novel *Pfdhps* mutants should be re-assessed.

**Supplementary Information:**

The online version contains supplementary material available at 10.1186/s12936-023-04487-5.

## Background

The threat of malaria continues to pose a major public health issue across the globe, specifically in sub-Saharan African countries, and especially among pregnant women and children under the age of five. In 2021 alone, the World Health Organization (WHO) reported that an estimated 247 million cases of malaria occurred worldwide, while the estimated number of deaths from malaria stood at 619,000 [1]. According to the report, approximately 27% of global malaria morbidity and 31.9% of mortality are attributed to Nigeria [1]. Currently, the WHO recommends chemopreventive strategies, including the use of sulfadoxine-pyrimethamine (SP) as intermittent preventive treatment during pregnancy (SP-IPTp), the use of SP (or other anti-malarials) as perennial malaria chemoprevention (PMC) and seasonal malaria chemoprevention (SMC) with amodiaquine and SP [2].

The SP-IPTp strategy, administered at fixed time points, at four-week intervals, during antenatal visits, is currently implemented in 33 sub-Saharan African countries [1]. It has been documented to ameliorate malarial illness and its associated risks both in maternal and fetal health outcomes in regions with medium to high malaria transmission [3]. The strategy is also considered one of the cost-effective malaria control tools available [4]. The effectiveness of SP-IPTp is measured by using metrics such as reduction in the incidence of maternal malaria infection, reduction in low birth weight (LBW), decrease in preterm delivery cases, and improved haematological parameters [4]. In Nigeria, the provision of SP-IPTp is recommended as part of antenatal care of all pregnant women [5]. The dosing schedule is typically set from the second trimester, and at least three doses are given before delivery, each at least one month apart.

The combination of sulfadoxine and pyrimethamine works synergistically, and preferentially, to target dihydropteroate synthetase (PfDHPS) and dihydrofolate reductase (PfDHFR), both enzymes involved in the folate biosynthesis pathway. Molecular mechanisms underpinning SP resistance are caused by single nucleotide polymorphisms (SNPs) in two distinct genes that codes for PfDHPS and PfDHFR, that is *Pfdhfr* and *Pfdhps* genes. Polymorphisms in codons of *Pfdhps* causing amino acid substitutions in PfDHPS at S/A436F, A437G, K540E, and A613S have been found to be associated with sulfadoxine resistance while SNPs in *Pfdhfr* causing amino acid changes at position 51, 59, 108 and 164 of PfDHFR confer pyrimethamine resistance [6, 7]. *Pfdhfr* triple mutant alleles resulting in changes N51I, C59R, S108N (**IRN**) and in *Pfdhps* gene, the A437G mutation is prevalent in West and Central African countries [8, 9]. In contrast, a quintuple mutant that combines the *Pfdhfr* triple (**IRN**) haplotype and the *Pfdhps* double mutant at A437G and K540E is highly prevalent in East Africa [6] while the *Pfdhps* K540E mutation is rarely found in West and Central Africa [10].

However, the narrative in East Africa has escalated in the last decade with the emergence of a SNP in *Pfdhps* causing an alanine to glycine mutation at position 581 of PfDHPS [4, 11]. This results in the ‘super resistant’ sextuple haplotype of *Pfdhfr*/*Pfdhps* (mainly the constructed haplotype: C**IRN**I-S**GEG**A). There is evidence that the C**IRN**I-S**GEG**A haplotype signals a loss of sensitivity of *Plasmodium falciparum* to SP and compromise the effectiveness of the SP-IPTp strategy including a reduction in its ability to prevent low birthweights [5, 6, 12, 13]. In contrast, in West Africa, the absence of 540 and 581 genotypes was reassuring for the continuation of SP-IPTp efficacy. However, recent reports have shown that the molecular repertoire of *P. falciparum* in West Africa may be changing. For instance, a SNP resulting in an isoleucine to valine change (*Pfdhps* I431V) has emerged in Nigeria and other parts of West Africa [14]. The mutant was first reported in the UK from a malaria infection imported from Nigeria [15] and it also appeared in other surveys conducted in Nigeria, and subsequently in Cameroon [16–18]. More recently, polymorphisms in *Pfdhps* resulting in amino acid substitutions at 431, 581 and 613 were reported in *P. falciparum* isolates from Ghana [19]. It has been suggested that the new mutant might disrupt sulfadoxine binding to its active site, thus reducing the susceptibility of *P. falciparu*m to the drug [14]. Considering the huge burden of malaria in Nigeria, particularly among pregnant women, and the lack of recent data on the extent of the spread of the *Pfdhps* 431V/581G mutation in the country and the region as a whole, there is an urgent need to evaluate the current prevalence of the *Pfdhps* 431V, *Pfdhps* 431V/-581G and *Pfdhps* 581G/-613S bearing parasites and, subsequently, the impact of these haplotypes on the protective efficacy of IPTp.

The present study assesses the prevalence of SNPs in *Pfdhps* in southwest Nigeria where the prevalence *Pfdhps* 431V/581G mutations are suspected to be increasing.

## Methods

### Study design

This was a cross-sectional study for sampling possible *P. falciparum* infection in pregnant women above 18 years who had received SP for IPTp and additionally, in both asymptomatic and febrile healthcare-seeking adults at a general outpatient clinic. The study participants were all tested for malaria by rapid diagnostic test (RDT) and those who tested positive were treated in line with the national guidelines for the treatment of uncomplicated malaria.

### Study area and site

The study was conducted at the Obafemi Awolowo University Teaching Hospitals Complex (OAUTHC) located in the Southwest region of Nigeria, at Ile-Ife in Osun State. Ile-Ife is situated in the Ife/Ijesa Senatorial district and the city has a population of approximately 400,763 people according to the last estimate of Nigeria’s population (https://populationstat.com/nigeria/ife). Ile-Ife stands between latitudes 7°28N and 7°45N, and longitudes 4°30E and 4°34E. The transmission of *P. falciparum* in this area is all-year-round [20]. The pregnant women were recruited at the Antenatal Clinic of OAUTHC, Ile-Ife. In addition, healthcare-seeking adults in the General Outpatient Department of the hospital also participated in the study.

### Ethics approval

Ethics approval was obtained from the Health Research and Ethics Committee (HREC) of the Obafemi Awolowo University Teaching Hospitals Complex, Ile-Ife (Protocol Number-ERC/2020/02/24). The prospective participants were provided with adequate information about the study and written informed consent was obtained from all the study participants eligible for participation.

### Sample collection

The samples were collected from the study participants between October 2020 and April 2021. Two millilitres of blood samples were collected in EDTA vacutainer tubes and 50 µl aliquots of each sample were spotted on 903 Whatman protein saver cards and stored as dried blood spot (DBS) samples. The DBS samples were stored with desiccant at room temperature prior to shipment to the Centre of Medical Parasitology, University of Copenhagen, for genomic analysis.

### DNA extraction

DNA was extracted in 1 ml DNA 96-well plates using DNA extraction kits supplied by Omega Bio-tek (Norcross, GA, USA). Each DBS sample was cut into the 1-ml well and the DNA was extracted according to the manufacturer’s manual. DNA samples were stored at − 20 °C until further downstream applications.

### Detection of *P. falciparum* infections

A nested PCR assay on the RDT positive samples for the detection of malaria parasite was performed as previously reported [21, 22]. The first PCR amplified the entire rPLU6 (TTAAAATTGTTGCAGTTAAAACG) and rPLU5 (CCTGTTGTTGCCTTAAACTTC). Primers for the nested PCR step were Plasm-all-n1fw (CCTTCAGTACCTTATGAGAAATC) and Plasm-all-n2rw (TCTGTCAATCCTACTCTTGTCTT). The cycle conditions for the primary PCR were: initial denaturation at 94 °C for 15 min followed by 30 cycles of annealing at 58 °C for 2 min, extension at 72 °C for 2 min, denaturation at 94 °C for 1 min with a final round of 58 °C for 2 min and 72 °C for 5 min. The conditions for the nested amplification were initial activation 94℃ for 15 min, denaturation at 94 °C for 1 min, annealing at 55 °C for 2 min, and extension at 72 °C for 2 min, with the last three steps repeated in 30 cycles. The amplification product from nested PCR was approximately 170 base pairs.

### Sequencing of molecular *Pfdhps* and *Pfdhfr* resistance markers

All samples positive for *P. falciparum* malaria were analysed using an Illumina targeted amplicon sequencing technique as described elsewhere with minor modifications [23].

### Data analysis

Sequencing data were analysed using Galaxy tools [24], trimmomatic [25], Python, and MS Excel (2016). Data were analysed with MS Excel and a plot was constructed with a plotting package in GraphPad^®^ software. The distributions of the haplotypes containing Pfdhps 431V allele among the two populations were compared using Fisher’s exact test. The difference was considered statistically significant if the P-value was less than 0.05.

### Mapping from previous reports on *Pfdhps* 431V, 437G, 540E, 581G and 613S resistant alleles

A survey of published reports since 2007 till date on the prevalence of *Pfdhps* 431V and 581G mutations in Nigeria [14, 16, 17, 26–35], and neighbouring countries [18, 36–47], was conducted, with no regards to age groups or specific population subgroups, to extract information on the total number of samples with mutations at the following positions 431, 437, 540, 581, 613, and the number of changes at each position. The mapping was performed in R version 4.0.3, and used shape files obtained from the Database of Global Administrative Areas Map (GADM) https://gadm.org/download_country.html.

## Results

A total of 561 participants consisting of pregnant women (n = 175) and healthcare-seeking adults (n = 386) gave their consent to donate 2 ml of venous blood, with a total of 188 samples (pregnant women (n = 40) and healthcare-seeking adults (n = 148)) being positive for malaria by RDT. However, assay by PCR revealed *P. falciparum* DNA in 128 samples out of 188 RDT positive samples. Only 33 (out of 40) samples among the asymptomatic pregnant women revealed malaria positivity after the PCR assay; 30 from the samples collected at enrolment during the third trimester and 3 collected at delivery (these were pooled together due a limited sample size during delivery). On the other hand, 88 (out of 148) samples among individuals recruited at the Outpatient Clinic were confirmed to be positive for malaria by PCR.

### Prevalence of *Pfdhps* and *Pfdhfr* haplotypes

All the PCR-based *P. falciparum* positive samples (n = 121) were genotyped for *Pfdhfr* and *Pfdhps*, out of which, 74 and 61 samples were successfully amplified, sequenced and fully profiled for mutations in *Pfdhfr* (50, 51, 59, 108 and 164) and *Pfdhps* (431, 436, 437, 540, 581 and 613)*,* respectively. A high prevalence of *Pfdhfr* mutant alleles (omitting mixed genotype infections) was detected at N51I (97.3%, 72/74), C59R (97.3%; 72/74) and S108N (98.6%; 73/74). The most common SNP in *Pfdhps* were the A437G identified in 100% of the samples. The prevalence of mutations at I431V, A581G and A613S were found to be 40.9% (25/61), 24.6% (15/61) and 42.6% (26/61), respectively, while no mutants were found at codon resulting in the amino acid substitution at K540E (Table 1). In addition, 49.2% of the samples (30/61) carried the 436 A allele (Table 1).

**Table 1:** The prevalence of *dhfr* and *dhps* SNPs in *P. falciparum* isolates from pregnant women and healthcare seeking adults in Ile-Ife, Southwest Nigeria

Gene	Group	SNP	Wild type n (%)	Mutant type n (%)	Mixed type n (%)
*Pfdhfr*	Pregnant women	N51I	0 (0)	16 (100)	0 (0)
		C59R	0 (0)	16 (100)	0 (0)
		S108N	0 (0)	16 (100)	0 (0)
		I164L	16 (100)	0 (0)	0 (0)
	Community	N51I	1 (1.7)	56 (96.6)	1 (1.7)
		C59R	1 (1.7)	56 (96.6)	1 (1.7)
		S108N	0 (0)	57 (98.3)	1 (1.7)
		I164L	58 (100)	0 (0)	0 (0)
	ALL	N51I	1 (1.4)	72 (97.2)	1 (1.4)
		C59R	1 (1.4)	72 (97.2)	1 (1.4)
		S108N	0 (0.0)	73 (98.6)	1 (1.4)
		I164L	74 (100.0)	0 (0.0)	0 (0.00)
*Pfdhps*	Pregnant women	I431V	11 (61.1)	6 (33.3)	1 (5.6)
		S436A	8 (44.4)	9 (50)	1 (5.6)
		A437G	0 (0)	18 (100)	0 (0)
		K540E	18 (100)	0 (0)	0 (0)
		A581G	16 (88.8)	1 (5.6)	1 (5.6)
		A613S	13 (72.2)	4 (22.2)	1 (5.6)
	Community	I431V	22 (51.2)	19 (44.2)	2 (4.6)
		S436A	17 (39.5)	21 (48.8)	5 (11.7)
		A437G	0 (0)	43 (100)	0 (0)
		K540E	43 (100)	0 (0)	0 (0)
		A581G	26 (60.5)	14 (32.6)	3 (6.9)
		A613S	18 (41.8)	22 (51.1)	3 (6.9)
	All	I431V	33 (54.1)	25 (40.9)	3 (4.9)
		S436A	25 (41.0)	30 (49.2)	6 (9.8)
		A437G	0 (0.0)	61 (100)	0 (0.0)
		K540E	61 (100)	0 (0.00)	0 (0.0)
		A581G	42 (68.9)	15 (24.6)	4 (6.6)
		A613S	31 (50.8)	26 (42.6)	4 (6.6)

Out of those successfully amplified and sequenced for *Pfdhps* and *Pfdhfr* genes, 61 and 74 of the *Pfdhps* and *Pfdhfr* sequences, respectively, were constructed into *Pfdhfr*/*Pfdhps* haplotypes, omitting infections with mixed genotypes at more than one codon (Table 2). For *Pfdhfr,* the triple mutant C50-**51I-59R-108N-**164I (C**IRN**I) haplotype was almost at fixation with a frequency of 95.8% (71/74). The other *Pfdhfr* haplotypes observed were the CN**RN**I (1.4%, n = 1) and **I**C**N**I (1.4%, n = 1). Eight distinct haplotypes of the *Pfdhps* gene were identified namely; IS**G**KAA, **V**A**G**KAA, **V**A**G**K**GS**, **V**A**G**KA**S**, IS**G**K**GS**, IA**G**KA**S**, IA**G**KAA and IA**G**K**GS** at a prevalence of 20 (32.8%), 9 (14.8%), 9 (14.8%), 6 (9.8%), 5 (8.2%), 4 (6.6%), 3 (4.9%) and 1 (1.6%), respectively. Combining *Pfdhfr* and *Pfdhps* haplotypes resulted in 10 discrete genotypes as presented in Table 2. The most abundant genotype was the quadruple mutants consisting of **IRN**I + IS**G**KAA (33.9%), **IRN**I + IA**G**KAA (5.4%) and N**RN**I + **V**A**G**KAA (1.8%). In the present data, three different sextuple mutant haplotypes existed, ranging from **IRN**I + IS**G**K**GS, IRN**I + IA**G**K**GS** to **IRN**I + **V**A**G**KA**S**, and all contributed 19.6% of the total haplotypic data. Another extensive mutant haplotype with 7 (septuple) mutant alleles was found in the isolates with genotypes **IRN**I + **V**A**G**K**GS** (16.1%).

**Table 2:** Genetic diversity of *Pfdhfr* and *Pfdhps* and the combined *Pfdhfr/dhps* haplotypes in *P. falciparum* isolates from Ile-Ife, Southwest Nigeria

Gene	Category of mutation	Haplotype	Community, n (%)	Pregnant, n (%)	Total, n (%)
*Pfdhfr* (n = 74)	Wild type	CNCSI	0 (0.0)	0 (0.0)	0 (0.0)
	Double	CN**RN**I	1 (1.7)	0 (0.0)	1 (1.4)
		C**I**C**N**I	1 (1.7)	0 (0.0)	1 (1.4)
	Triple	C**IRN**I	55 (94.9)	16 (100.0)	71 (95.8)
*Pfdhps* (n = 61)	Wild type	ISAKAA	0 (0.0)	0 (0.0)	0 (0.0)
	Single	IS**G**KAA	12 (27.9)	8 (44.4)	20 (32.8)
		IA**G**KAA	2 (4.7)	1 (5.6)	3 (5.8)
	Double	IA**G**KA**S**	2 (4.7)	2 (11.1)	4 (6.6)
		**V**A**G**KAA	5 (11.6)	4 (22.2)	5 (9.62)
	Triple	IS**G**K**GS**	5 (11.6)	0 (0.0)	9 (14.8)
		IA**G**K**GS**	1 (2.3)	0 (0.0)	1 (1.6)
		**V**A**G**KA**S**	5 (11.6)	1 (5.6)	6 (9.8)
	Quardruple	**V**A**G**K**GS**	8 (18.6)	1 (5.6)	9 (14.8)
*Pfdhfr*/*Pfdhps* (n = 56)	Quadruple mutant	**IRN**I + IS**G**KAA	11 (28.2)	8 (47.1)	19 (33.9)
		**IRN**I + IA**G**KAA	2 (5.1)	1 (5.9)	3 (5.4)
		N**RN**I + **V**A**G**KAA	1 (2.6)	0 (0.0)	1 (1.8)
	Quintuple mutant	**I**C**N**I + **V**A**G**KA**S**	1 (2.6)	0 (0.0)	1(1.8)
		**IRN**I + IA**G**KA**S**	2 (5.1)	2 (11.8)	4 (7.2)
		**IRN**I + **V**A**G**KAA	4 (10.3)	4 (23.5)	8 (14.2)
	Sextuple mutant	**IRN**I + IS**G**K**GS**	5 (12.8)	0 (0.0)	5 (8.9)
		**IRN**I + IA**G**K**GS**	1 (2.6)	0 (0.0)	1 (1.8)
		**IRN**I + **V**A**G**KA**S**	4 (10.3)	1 (5.9)	5 (8.9)
	Septuple mutant	**IRN**I + **V**A**G**K**GS**	8 (20.5)	1 (5.9)	9 (16.1)

**Fig. 1:**
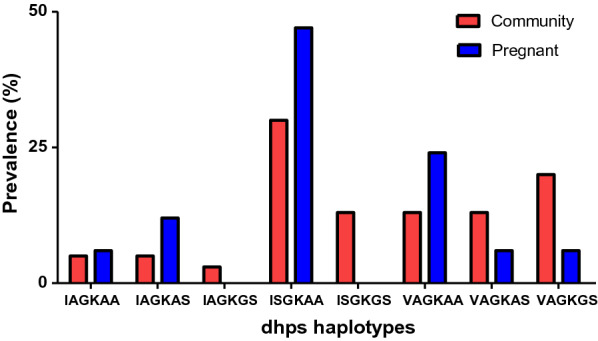
Bar Chart comparing the prevalence of dhps haplotypes among pregnant women and community setting (N_pregnant women_ = 17, N_community_ = 40)

The frequency of the *Pfdhfr* I**RNI** haplotype was as expected, widespread in both pregnant women and in the community arm. The prevalence of the haplotypes that contained 431V mutation (**V**A**G**KAA, **V**A**G**KA**S**, **V**A**G**K**GS**) appeared not to be statistically significant between the two groups (Fisher’s exact test, P-value = 0.568) (Fig. 1).

### Current knowledge on the distribution of *Pfdhps* genotypes in Nigeria and the neighbouring countries

**Fig. 2:**
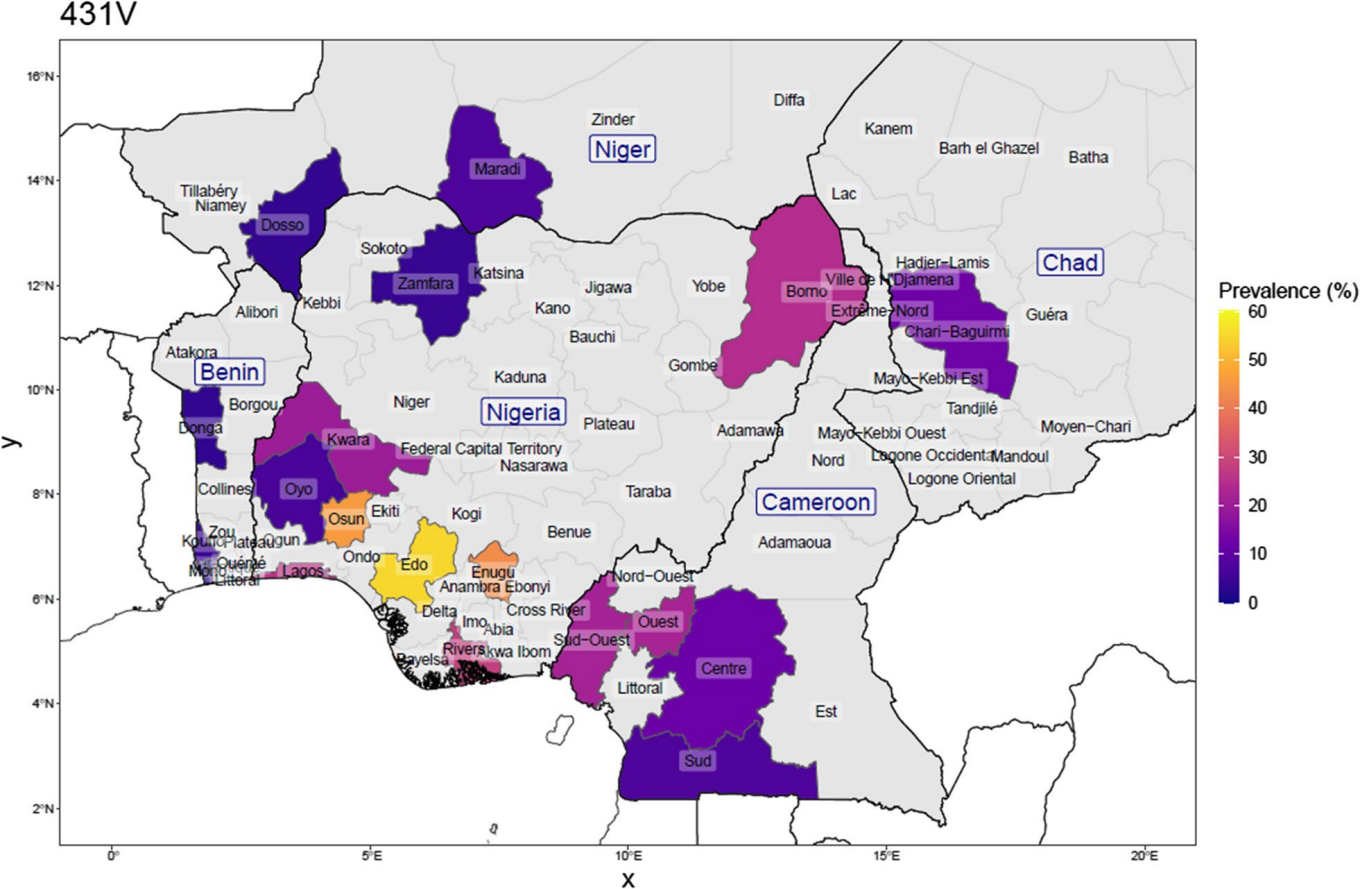
Prevalence of the *Pfdhps*-431V mutation in Nigeria, and bordering countries by upper administrative unit, using published studies of surveys done between 2007 and 2021 (for Nigeria: 25 surveys from 16 studies; N_total samples_ = 2262, and for neighbouring countries: 32 surveys from 27 studies; N_total samples_ = 3248). This is calculated as the number with the mutation among the number of samples determined at that position. Proportions were weighed by each study’s sample size for regions where more than one study was conducted

**Fig. 3:**
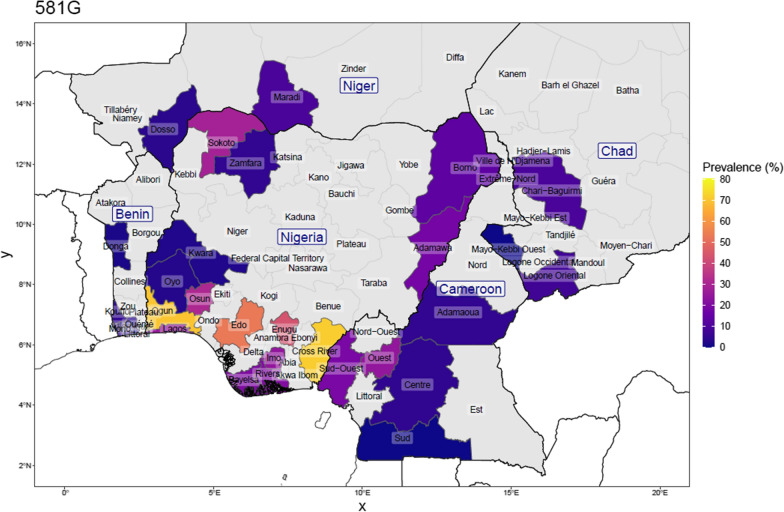
Prevalence of the dhps-581G mutation in Nigeria, and bordering countries by upper administrative unit, using published studies of surveys done between 2007 and 2021 (for Nigeria: 25 surveys from 16 studies; N_total samples_ = 2609, and for neighbouring countries: 32 surveys from 27 studies; N_total samples_ = 5705). This is calculated as the number with the mutation among the number of samples determined at that position. Proportions were weighed by each study’s sample size for regions where more than one study was conducted

**Fig. 4:**
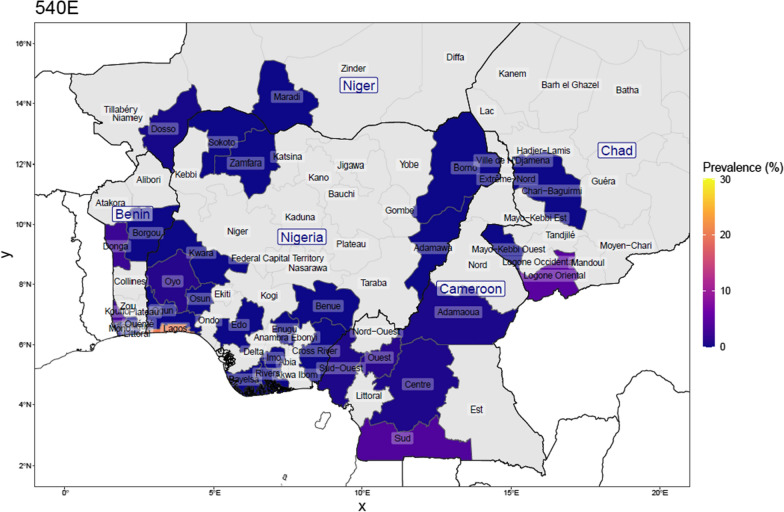
Prevalence of the dhps-540E mutation in Nigeria, and bordering countries by upper administrative unit, using published studies of surveys done between 2007 and 2021 (for Nigeria: 25 surveys from 16 studies; N_total samples_ = 2869, and for neighbouring countries: 32 surveys from 27 studies; N_total samples_ = 6519). This is calculated as the number with the mutation among the number of samples determined at that position. Proportions were weighed by each study’s sample size for regions where more than one study was conducted

**Fig. 5:**
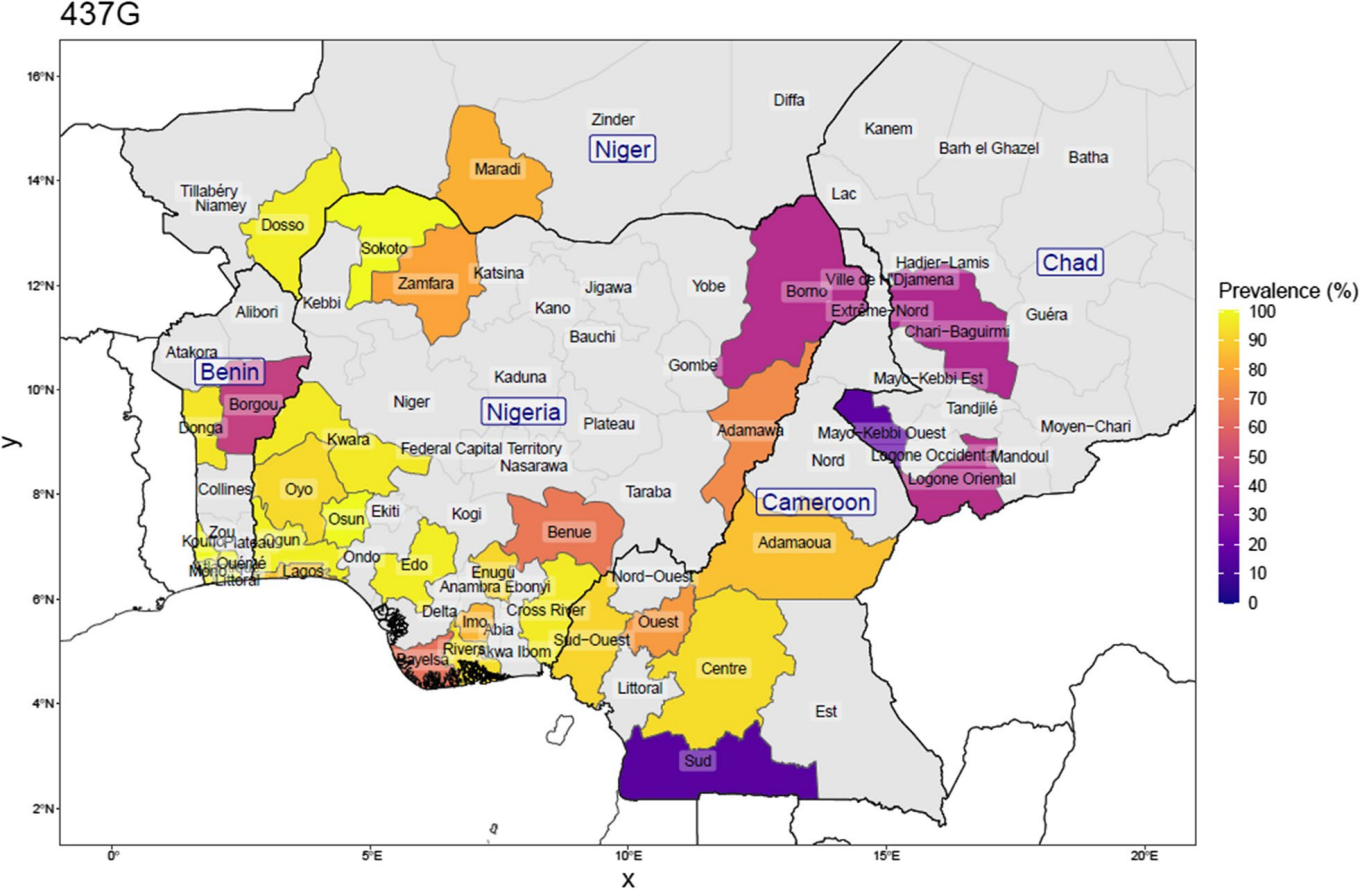
Prevalence of the dhps-437G mutation in Nigeria, and bordering countries by upper administrative unit, using published studies of surveys done between 2007 and 2021 (for Nigeria: 25 surveys from 16 studies; N_total samples_ = 2794, and for neighbouring countries: 32 surveys from 27 studies; N_total samples_ = 6587). This is calculated as the number with the mutation among the number of samples determined at that position. Proportions were weighed by each study’s sample size for regions where more than one study was conducted

**Fig. 6:**
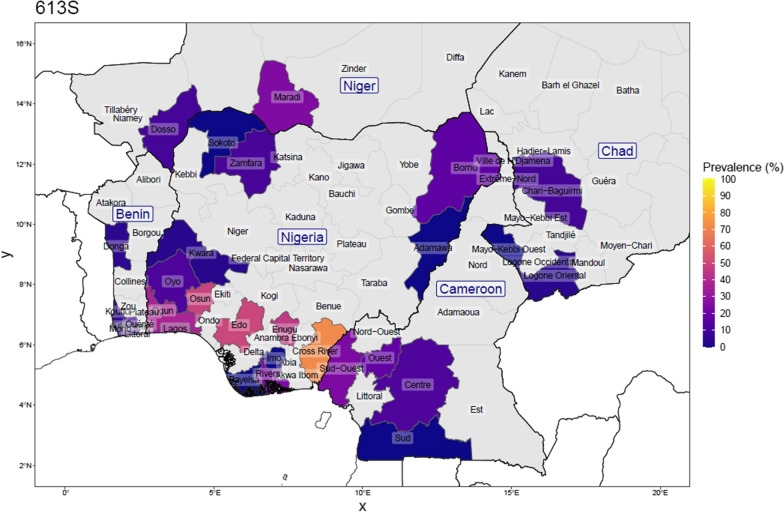
Prevalence of the dhps-613S mutation in Nigeria, and bordering countries by upper administrative unit, using published studies of surveys done between 2007 and 2021(for Nigeria: 25 surveys from 16 studies; N_total samples_ = 2606, and for neighbouring countries: 32 surveys from 27 studies; N_total samples_ = 3936). This is calculated as the number with the mutation among the number of samples determined at that position. Proportions were weighed by each study’s sample size for regions where more than one study was conducted

Genotype data from the literature spanning surveys done between 2007 and 2020, were combined with the genotype data from the current study to assess the current distribution of *Pfdhps* mutations, and to briefly describe the patterns observed (Figs. 2, 3, 4, 5, 6). Published surveys (Additional file 1: Review of surveys on dhps mutation in Nigeria and neighbouring countries) of *Pfdhps* 431V were limited to only 9 States in Nigeria whereas those of other *Pfdhps* SNPs had wider coverage (15 States). The *Pfdhps* 431V mutation reports, show that it has already reached a prevalence of 54.0% in Edo, closely followed by Enugu (43.3%) as well as Osun (45.9%) in the present study. The *Pfdhps* 431V allele has also been reported in the surveys conducted in Borno (24.5%), Kwara (20.0%), and Lagos (25.9%), Oyo (6.6%), and Rivers (28.4%) and Zamfara (4.0%) states. The data on *Pfdhps* 437G mutation gathered from published studies revealed that it is widespread throughout Nigeria (prevalence ranging from 41.4 to 100%). Borno state in the Northeast region being the only area where the prevalence of *Pfdhps* 437G was less than 50%. The mutation has already attained 100% in most places in Nigeria. By contrast, the surveys reporting on *Pfdhps* 540E (Fig. 4) showed that the mutation is very rare in Nigeria. Of the fifteen states where published surveys were conducted, *Pfdhps* 540E was detected only in Lagos state (20.1%) and Oyo state (1.0%), and it appears to be non-existent in other states. Variations in the prevalence of *Pfdhps*-581G and -613S were observed across the country and broadly there is a geographical division between a high prevalence of the *Pfdhps* 581G/613S (Figs. 5, 6) in the southern States compared to northern states. A much higher prevalence of *Pfdhps* 581G was observed in Ogun and Cross-River States reaching 69.8 and 71.4%. Some other States in the South-south and Southeast showed a prevalence between 20 and 50%.

Considering the studies from four neighbouring countries, the prevalence of SP resistance markers was reported in 10 surveys in Benin, in 14 surveys in Cameroon, in 3 surveys in Chad and in 5 surveys in Niger. The samples collected in Cameroon pre-2011 had no 431V and 581G mutation. The *Pfdhps* 540E mutation was also rarely found in the four neighbouring countries (0.75%). Among the samples reported in all the surveys carried out in Cameroon, 16.6%, 8.5% and 17.7% carried the *Pfdhps*-431V, -581G and -613S mutants, respectively. In Benin, the data from the 10 surveys indicate 6.8%, 5.9% and 9.7% prevalence of the *Pfdhps*-431V, -581G and -613S mutations, respectively. Both Chad and Niger Republic survey data indicated a similar trend.

## Discussion

The WHO encourages the use of SP-IPTp as part of antenatal care in malaria-endemic settings in Africa. In this study, asymptomatic falciparum malaria was found in 19% of pregnant women. Previous reports on asymptomatic malaria in pregnancy, have shown asymptomatic malaria in pregnancy is prevalent across sub-Saharan African (SSA) countries, and this carries an increased risk of maternal anaemia compared to non-infected pregnant women [48, 49]. It has been well established that asymptomatic malaria infection is associated with poor maternal or fetal health [48]. SP resistance is known to modify the outcome of SP-IPTp strategy. The occurrence of maternal and placental malaria among pregnant women who received SP-IPT may be linked to reduced drug susceptibility of the malaria parasites due to SP resistance as a result of emergence of SNPs in the *Pfdhfr*/*Pfdhps* genes of *P. falciparum* [4, 11, 50–52].

Findings from this study showed that the SNPs in *Pfdhfr* N51**I**, -C59**R**, -S108**N** (*Pfdhfr* haplotype C**IRN**I) and *Pfdhps*-A437**G** are widespread in the studied population, reaching a prevalence of beyond 95%. Similar findings have been reported in Nigeria and other countries across SSA [17, 53]. This suggests that the quadruple mutations are already fixed in these populations [18, 32, 54]. Indications from other studies however revealed that the protective effect of SP-IPTp remains intact, regardless of the high prevalence of this quadruple *Pfdhfr*-*Pfdhps* mutant [2, 55].

However, the emerging of certain *Pfdhps* mutant haplotypes in the West African region raise concerns about the continuing effectiveness of SP-IPTp. The present study has substantiated findings from previous studies that suggested the emergence of other *Pfdhfr/Pfdhps* mutant alleles in Nigeria and the West African region. The present study showed that some *P. falciparum* isolates were carrying additional mutations at codons I431V, A581G and A613S particularly in the South. However, the frequency of these new mutations is not fixed and furthermore it is not clear how far the new mutations will influence the efficacy of SP-IPTp (and possibly other preventive interventions using SP alone; e.g. the newly revised Perennial Malaria Chemoprevention of infants). Before the advent of the *Pfdhps* 431V mutation in Nigeria, East Africa witnessed a similar scenario with the emergence of the quintuple (**IRN** + 437**G** + 540**E**) and sextuple (**IRN** + 437**G** + 540**E** + 581**G**) mutant haplotypes. These were associated with a reduction in the mean birth weight and other metrics of SP-IPTp failure in Tanzania [13, 56]. Although no 540E mutation was observed in the present study, it is possible that the emerging *Pfdhps* 431V and its co-occurrence with 581G and 613S could lead to a higher order mutant haplotype, and this alternative sextuple mutant might compromise the chemopreventive impact of SP-IPTp in West Africa.

Previous studies indicated that the prevalence of *Pfdhps* 431V varies across regions in Nigeria in clinical isolates collected at various respective study periods [13–15]. For instance, a low prevalence of *Pfdhps* 431V mutation was reported in Southwest Nigeria, whereas the prevalence in the parasite population is widespread in the Southeast and Northeast Nigeria among the samples collected about a decade ago [13]. Figures 2 and 3 give a view of the spatial distribution of *Pfdhps*-431V and -581G in Nigeria and neighbouring countries, using data reported in surveys conducted between 2007 and 2020. This study was the first survey in Osun State and it revealed a high prevalence of the *Pfdhps*-431V (45.9%) and -581G (31.1%) mutations. The *Pfdhps*-431V mutation is found to be higher than compared to the neighbouring states, both Oyo and Kwara. Considering the upsurge of mutation in the present study, it would be necessary to bear in mind that the data for Oyo/Kwara are mostly based on older surveys. Oguike et al. [14] provided data in a study site located in Ibadan, approximately 100 km from the present study site, with a frequency of 0 and then,6.5% from a sequential sampling design between 2003 and 2008. There is a high possibility that the level of *Pfdhps* mutant alleles might have also increased in all the surrounding states. A more recent report by Oboh et al. [16] in Lagos and Edo States revealed a prevalence of 28.5% and 51.4%, respectively, in clinical samples collected between 2016 and 2017 [16]. This is in support that the prevalence of *Pfdhps* 431V mutation is increasing across the Nigerian population. This present data revealed a frequency of 31.1% for the dhps-581G bearing parasites. This also suggests an upsurge in the circulating 581G mutant allele in Southwest Nigeria similar to previous findings from Lagos and Ogun States [14, 16]. The SNPs A581G and I431V co-occur in 21.3% of the parasite population in this study. This was suggested to render the parasite less susceptible to sulfadoxine [17]. However, the paradigm is already changing to a higher order mutant allele combination consisting the Nigerian sextuple or septuple mutant haplotypes.

It is worth noting that eight distinct haplotypes were detected on the *Pfdhps* gene starting from IS**G**KAA, a single mutation, to **V**A**G**K**GS** with 4 mutant alleles. Other haplotypes were IA**G**KAA, IA**G**KA**S**, IS**G**K**GS**, **V**A**G**KAA, IA**G**K**GS** and **V**A**G**KA**S.** The wild haplotype ISAKAA was not found in this study in contrast to the findings reported by Oboh et al. [16]. Most haplotypes were present among the study populations except IS**G**K**GS** and I**AG**K**GS** which were only present in the group seeking healthcare at the Outpatient Unit. An important observation from this study is a relatively high prevalence of sextuple and septuple haplotypes when the SNPs from both *Pfdhfr* and *Pfdhps* genes were combined. Both haplotypes contributed approximately 25.0% of the *P. falciparum* isolates in the present study, and their presence might pose a high probability of the malaria parasite becoming extensively resistant to SP in Nigeria.

Despite the limitations of the study, such as the small sample size and the use of only one study site, the present data provides information about the current prevalence of molecular markers of SP resistance in Southwest Nigeria. The study revealed that a high frequency of the emerging mutations now exists within the region. In previous studies, other regions in Nigeria depicted a much higher frequency of *Pfdhps* 431V. Thus, the results of the present study, conducted in the Southwest region of the country, suggest a need to reassess the current prevalence of *Pfdhps* mutation in other regions as the current prevalence in those regions might have reached an alarming proportion. However, the exact implication of an increase in the frequency of mutation has not been established. Therefore, further work is suggested to examine in vivo susceptibility of the mutant malaria parasites to SP and evaluate the efficacy of SP-IPTp in the context of the emerging mutations.

## Supplementary Information

**Additional file 1.** Review of surveys on dhps mutation in Nigeria and neighbouring countries.

## Data Availability

Data are available upon reasonable request to the corresponding author.

## Abbreviations

SP-IPTp: Sulfadoxine-Pyrimethamine for Intermittent preventive treatment during pregnancy
Pfdhps: *Plasmodium falciparum* Dihydropteroate synthetase
Pfdhfr: *Plasmodium falciparum* Dihydrofolate reductase
PCR: Polymerase chain reaction
PMC: Perennial malaria chemoprevention
SMC: Seasonal malaria chemoprevention
SNPs: Single nucleotide polymorphisms
RDT: Rapid diagnostic test
OAUTHC: Obafemi Awolowo University Teaching Hospitals Complex
HREC: Health Research and Ethics Committee
DBS: Dried blood spot
GADM: The Database of Global Administrative Areas Map (GADM)
SSA: Sub-Saharan African
WHO: World Health Organization
